# Novel Mechano-Luminescent Sensors Based on Piezoelectric/Electroluminescent Composites

**DOI:** 10.3390/s110403962

**Published:** 2011-04-01

**Authors:** Yanmin Jia, Xiangling Tian, Zheng Wu, Xiaojuan Tian, Jiayi Zhou, Yunzhang Fang, Chenchen Zhu

**Affiliations:** 1 Department of Physics, Zhejiang Normal University, Jinhua, 321004, China; E-Mails: willy111999@163.com (X.T.); tudouboshi@126.com (X.T.); zhoujiayi28@126.com (J.Z.); Fangyz@zjnu.cn (Y.F.); 2 College of Geography and Environmental Sciences, Zhejiang Normal University, Jinhua, 321004, China; E-Mail: wuzheng@zjnu.cn (Z.W.); 3 School of Materials Science and Technology, China University of Geosciences, Beijing, 100083, China; 4 ACCIS Group, Aerospace Engineering, Faculty of Engineering, University of Bristol, Tyndall Avenue, Bristol BS8 1TH, UK; E-Mail: zcc65@hotmail.com (C.Z.)

**Keywords:** piezoelectric, mechano-luminescent sensors, composites

## Abstract

A high-sensitivity mechano-luminescent sensor was fabricated on the basis of piezoelectric/electroluminescent composites. The working principle of this mechano-luminescent sensor was elucidated by analyzing the relationship between the piezoelectric-induced charges and the electroluminescent effects. When a stress is applied on the piezoelectric layer, electrical charges will be induced at both the top and bottom sides of the piezoelectric layer. The induced electrical charges will lead to a light output from the electroluminescent layer, thus producing a mechano-luminescence effect. By increasing the vibration strength or frequency applied, the mechano-luminescence output can be obviously enhanced. Mechano-luminescence sensors have potential in smart stress-to-light devices, such as foot-stress-distribution-diagnosis systems and dynamic-load-monitors for bridge hanging cables.

## 1. Introduction

The mechano-luminescent effect is a light emission induced as a result of some mechanical action on a solid [1–4]. In 1605, Francis Bacon noted that lumps of sugar emitted light when scraped [5]. The mechano-luminescent effect can be produced through either destructive means (grinding, rubbing, scratching, biting, cleaving) or non-destructive means (ultrasound, stress, vibration or through other means) [6]. Until now, destructive mechano-luminescent effects have been extensively observed in various inorganic and organic materials [7–11]. However, due to the non-repeatability of destructive mechano-luminescent response, it is difficult to obtain practical devices based on application of destructive mechano-luminescent effects [12,13].

Non-destructive mechano-luminescent materials are promising for practical stress sensor applications [14–16], such as foot-stress-distribution-diagnosis systems, stress-light devices, and monitoring dynamic load for bridge hanging cables. However, due to the rigorous demands of the mechano-luminescent effect with regards to crystal structure symmetry, non-destructive mechano-luminescent materials have only been found in a few limited cases [12,13]. On the other hand, in most of the stress-luminescent materials reported, the coupling between stress and luminescence was very weak and occurred only under low-temperature conditions [13,17]. Hence, so far, no practical application of mechano-luminescent has been realized. Exploration of high performance non-destructive mechano-luminescent materials is a necessary precondition for designing mechanical-optical smart sensor devices.

It’s well known that the product effect method can be used to realize a certain property which does not exist in either of the individual phases. In a biphasic combination material, if one phase possesses a property A → B (use of an independent variable property A leading to a property B) with a constant ratio tensor *dB*/*dA* = *X*, and the other phase displays a property B → C with a constant ratio tensor *dC*/*dB* = *Y*, thus the combination material will exhibit a property A → C which is unavailable in either of the initial phases. The product effect method can help us to accomplish strong coupling between two optional physical signals (optical, thermal, electrical, mechanical, and magnetic) by fabricating suitable composites. A typical example is the giant magnetoelectric effect observed in a magnetostrictive/piezoelectric composite such as Tb_x_Dy_1−x_Fe_2_ and (1−*x*)Pb(Mg_1/3_Nb_2/3_)O_3_−*x*PbTiO_3_ (PMN-PT) [18,19]. An alternating magnetic field can induce a strain in the magnetostrictive phase. The strain will distort the piezoelectric phase to generate electric charges. Thus a strong coupling between the magnetic and electrical signals can be realized. On the basis of the product effect principle, we tried to fabricate a high mechano-luminescent performance composite for smart mechanical-optical sensor devices by using the product-composite method.

In this article, we report the presence of a high mechano-luminescent effect obtained by using the product of a piezoelectric effect and an electroluminescent effect. Piezoelectric materials have the excellent property of converting force (stress) signals into electrical charge (or voltage) signals. Under an alternating electric field, electroluminescent materials can directly convert electric energy to light. On the basis of the principle of product effects [18,19], a strong stress-luminescent effect could be accomplished by fabricating piezoelectric/electroluminescent composites. Such composites with high mechano-luminescent performance have potential for application in smart and multifunctional devices, such as artificial skin, smart skin, self-diagnosis systems or stress-distribution imaging systems.

## 2. Experimental Section

**Figure 1.**
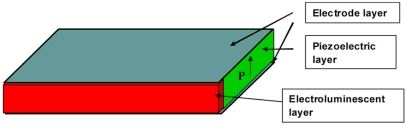
The geometry of the mechano-luminescent composite.

Figure 1 illustrates the geometry of the mechano-luminescent composite under investigation. Our composite consists of one piezoelectric PMN-PT crystal layer polarized along the thickness direction and one electroluminescent layer. The PMN–PT plates, of 12 × 6 × 1 mm^3^ dimensions, and with the <001> and <011> crystallographic axes oriented along the length and thickness directions, respectively, were grown in-house using a modified Bridgman technique [20]. It was shown in our previous work that PMN–PT plates, when grown with these specific crystallographic orientations and polarized using the <001> axis along the thickness direction, possess an ultrahigh transverse piezoelectric response with deformations along the length direction when excited electrically along the thickness direction [21]. After being electroded with silver and polarized along the thickness direction in silicone oil, the piezoelectric coefficient (*d*_31,p_) and elastic compliance coefficient $\left(s_{11}^{E}\right)$ of the PMN-PT plates were measured to be −1,300 pC/N and 126 pm^2^/N, respectively, using an Agilent 4294A impedance analyzer following the IEEE resonance method [22]. The electroluminescent layer was commercially supplied Surface Mounted Devices (SMD) red light emitting diodes. The piezoelectric layer was bonded together with an electroluminescent layer using an insulating epoxy, as is shown in Figure 1. Two electrode layers (conductive Ag glue) were laid down to maintain a solid electrical connection between the piezoelectric and electroluminescent layers.

The working principle of this sensor can be described as follows: when we apply a stress to the piezoelectric layer, electrical charges will be induced at both the top and bottom faces of piezoelectric layer due to the piezoelectric effect. These induced electrical charges will result in a light output from the electroluminescent layer due to the electroluminescent effect. A signal coupling between stress and light is thus accomplished.

The mechano-luminescent effect in the fabricated composites was characterized using an in-house automated measurement system. In our experiments, we adopted an ultrasonic vibration machine (Model KQ-100VDE, Kunshan Ultrasonic Equipment Co., Ltd.; frequency 28 kHz, 45 kHz, 100 kHz; power: 40 ∼ 100 W) to apply a mechanical signal. This method has been used in several references [4,23]. The samples for measurement were put into a glass beaker filled with an electrically insulating and transparent silicone oil. The glass beaker was put into the ultrasonic vibration source. The voltage output from the piezoelectric layer was measured using an oscilloscope. The luminescent output of the samples was measured by Andor’s Mechelle ME5000 spectrograph (wavelength range 200 ∼ 975 nm). The mechano-luminescent output at various vibration strengths and frequencies was studied systematically.

## 3. Results and Discussion

**Figure 2.**
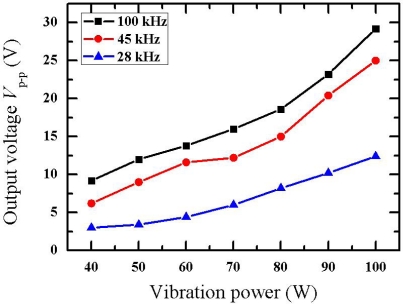
The piezoelectric voltage output as a function of ultrasonic vibration power at 28 kHz, 45 kHz and 100 kHz, respectively.

Figure 2 shows the piezoelectric voltage output as a function of ultrasonic vibration power at 28 kHz, 45 kHz and 100 kHz, respectively. In the measurement range (40 ∼ 100 W), the output voltage *V*_P-P_ ranged from 3 V to 29.2 V. The high voltage output makes it possible to drive the electroluminescent layer. As the vibration power or frequency increased, the piezoelectric voltage output went up correspondingly. The maximum voltage output *V*_P-P_ ∼ 29.2 V occurred at an ultrasonic vibration power 100 W and a frequency of 100 kHz.

We can estimate the practical force applied on the sample as follows:

(1) Q=dT

(2) Q=CU

where *Q* is the electrical charge from piezoelectric phase’s surface. *d* is the piezoelectric charge coefficient. *T* is the force applied on the piezoelectric layer. *C* is the electrical capacitance of piezoelectric phase. From Equations (1) and (2), we can obtain:

(3) T=CU/d

For the PMN-PT piezoelectric crystal, the parameter *C* is about 3 nF, and the parameter *d* is about 1,500 pC/N. The force applied on the sample is in the range of 2 ∼ 30 N, which can be calculated by substituting the peark voltage value of 2 ∼ 15 V from Figure 2 into Equation (3).

**Figure 3.**
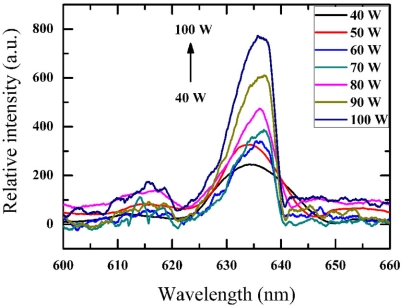
The mechano-luminescent output spectrograph of the composite under different vibration powers.

The mechano-luminescent light output (red light) can be clearly observed by the naked eye while vibrating the composite in silicone oil by using ultrasonic waves at room temperature. Figure 3 shows the stress-luminescent output spectrograph of the composite under different vibration powers. The wavelength of ∼635 nm corresponds to red light. The brightness increased with increasing piezoelectric vibration power (at 45 kHz). It has been reported that electroluminescent brightness increases with the increase of applied voltage [24].

On the basis of empirical and theoretical results, the voltage dependence of the electroluminescent brightness or emission intensity may be rendered as [25]:

(4) $$B=a_{0}f^{1/2}\;\text{exp}\left(-b_{0}/\sqrt{V}\right)$$

where *a*_0_ and *b*_0_ are constants, which depend on a given temperature, the frequency of the applied voltage and the size of electroluminescent samples and *f* is the frequency. The dependence of electroluminescent brightness on the square root of applied voltage is normally explained on the basis of an electron acceleration-collision mechanism [26].

**Figure 4.**
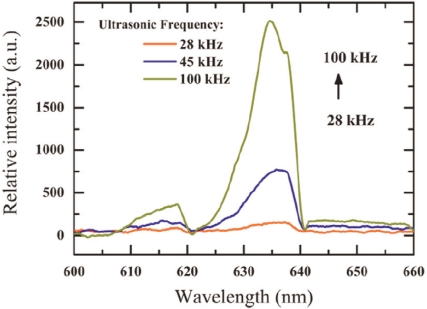
The mechano-luminescent output spectrograph of the composite under frequencies of 28 kHz, 45 kHz and 100 kHz, respectively.

Figure 4 shows the mechano-luminescent output spectrograph of the composite under frequencies of 28 kHz, 45 kHz and 100 kHz, respectively (at 100 W vibration power). With the increase of frequency, the electroluminescent brightness obviously went up as well. The frequency response of electroluminescent materials can be accounted for on the basis of Equation (1). It should be noted that the wavelength peaks shift from 637 nm to 634 nm with the frequency going up from 28 kHz to 100 kHz, *i.e*., a small “blue-shift” occurs as the frequency rises. This sis due to the energy levels’ emission (red emitters), which becomes higher with higher frequency alternating vibration [27,28].

On basis of the structure of this composite, piezoelectric and electroluminescent effect also exist in this stress-light laminate. Thus the mechano-luminescent composite can also be used as a self-powered electroluminescent or photoluminescent sensor. Due to the fact that piezoelectric materials and ac electroluminescent materials only respond to dynamic signals, the applied mechanical signals on the novel sensors must be continuous and non-destructive in practical application. What’s more, the amplitude of the applied stress should be moderate (∼N to ∼kN). Too strong a stress might damage the mechano-luminescent sensors. Converting weak mechanical stress to luminescence efficiently is still a question. We hope to further enhance the sensitivity of mechano-luminescent sensors by adopting better piezoelectric and electroluminescent materials, strengthening the interface coupling between the two phases and optimizing the working circumstances such as temperature and humidity.

The mechano-luminescent effect in this composite can directly convert mechanical signals into light. It is helpful to measure the distribution of mechanical stress over an area. The mechano-luminescent composites have the potential for application in smart sensor devices, such as foot-stress-distribution-diagnosis systems, stress-distribution imaging systems, artificial skin, self-diagnosis systems, dynamic-load-monitor for bridge hanging cables and other stress-light devices.

## 4. Conclusions

In summary, a giant stress-luminescent effect was generated by fabricating a piezoelectric/electroluminescent composite. Red-light emission was visible to the naked eye, when a stress of 2 ∼ 15 N was applied on this composite. Increasing the vibration’s strength or frequency can obviously enhance the mechano-luminescent output. A small “blue-shift” occurs as the frequency rises due to the high energy levels’ emission at high vibration frequencies. These strong mechano-luminescent materials are promising for applications such as novel stress sensors and displays to visualize stress distributions.
